# Biliary Tract and Pancreatic Cancer (BTPC) in Adult Patients: The Role of the Biliary Microbiota in Cancer and Therapeutic Strategies—A Scoping Review

**DOI:** 10.3390/cancers18121875

**Published:** 2026-06-08

**Authors:** Paola Di Carlo, Nicola Serra, Aducio Thiesen, Vito Rodolico, Antonio Cascio, Teresa Maria Assunta Fasciana, Anna Giammanco, Valentina Caputo, Gianfranco Cocorullo, Giuseppe Salamone, Giuseppe Carollo, Consolato M. Sergi

**Affiliations:** 1 Department of Health Promotion, Maternal-Childhood, Internal Medicine of Excellence G. D’Alessandro, Section of Infectious Disease, University of Palermo, 90127 Palermo, Italy; paola.dicarlo@unipa.it (P.D.C.); vito.rodolico@unipa.it (V.R.); antonio.cascio03@unipa.it (A.C.); teresa.fasciana@unipa.it (T.M.A.F.); valentina.caputo@unipa.it (V.C.); 2 Department of Neuroscience, Reproductive Sciences and Dentistry Department, Audiology Section, University of Naples Federico II, 80131 Naples, Italy; 3 Department of Laboratory Medicine and Pathology, University of Alberta, Edmonton, AB T6G 2R3, Canada; aducio.thiesen@albertaprecisionlabs.ca (A.T.); sergi@ualberta.ca (C.M.S.); 4 Legionella Reference Laboratory, University of Palermo, 90127 Palermo, Italy; anna.giammanco@unipa.it; 5 Department of Precision Medicine in Medical, Surgical and Critical Care (Me.Pre.C.C.), University of Palermo, 90127 Palermo, Italy; gianfranco.cocorullo@unipa.it (G.C.); giuseppe.salamone@unipa.it (G.S.); giuseppe.carollo@policlinico.pa.it (G.C.)

**Keywords:** biliary microbiome, dysbiosis, biliary tract cancer, pancreatic cancer, precancerous lesions, chronic inflammation, chemoprophylaxis, biliary stenting, microbiota, carcinogenesis

## Abstract

**Background:** The biliary and pancreatic tract is increasingly recognized as a microbial ecosystem rather than a sterile environment. Dysbiosis contributes to inflammation, bile acid alterations, and carcinogenesis, with distinct microbiota profiles linked to progression from benign to malignant conditions. Clinical factors, including gut–liver axis disruption and biliary stenting, may further exacerbate microbial imbalance. **Objective:** The objective of this study is to synthesize current evidence and identify knowledge gaps on the role of biliary microbiota in pancreaticobiliary carcinogenesis and its implications for diagnosis, prognosis, and therapy. **Methods:** This scoping review was conducted following PRISMA-ScR guidelines. A systematic search of PubMed, Web of Science, and Scopus was performed for studies published between January 2015 and December 2025, guided by the PICo framework. **Results:** Included studies primarily characterized changes in microbiota composition to identify microbial biomarkers associated with pancreaticobiliary diseases. Predictive bioinformatics analyses suggest that dysbiosis may promote carcinogenesis through metabolic and inflammatory pathways. Machine learning approaches identified microbiota-based signatures with potential diagnostic value for precancerous lesions, although discrimination remains limited. Biliary dysbiosis was also associated with outcomes related to biliary stenting, chemoprophylaxis, postoperative complications, and responses to chemotherapy or surgery. **Conclusions:** Integration of microbiota profiling with predictive bioinformatics and machine learning may improve understanding of pancreaticobiliary carcinogenesis. Identifying microbial and functional biomarkers could enable personalized diagnostic and therapeutic strategies, ultimately improving patient outcomes.

## Simple Summary

This scoping review traces the evolution of biliary microbiota research, driven by the application of the 16S technique and advanced bioinformatics analyses, which have enabled the identification of specific microbial signatures correlated with biliary neoplasms. In this context, the authors aim to evaluate whether the microbiome associated with biliary–pancreatic tumors could serve as a promising biomarker for the early diagnosis of pancreatic tract neoplasms. While acknowledging the intrinsic variability of the microbiome among individuals, the potential benefits in terms of concrete clinical implications, including from a pharmacological perspective, are emphasized. Indeed, the intestinal microbiota can influence the onset and progression of cancer, as well as the efficacy and toxicity of chemotherapy, radiotherapy, and immunotherapy.

## 1. Introduction

The biliary and pancreatic systems, encompassing both intrahepatic and extrahepatic ducts as well as the gallbladder, common bile duct, and pancreatic ducts, were traditionally regarded as sterile; however, they are now recognized as environments hosting a complex microbiota whose roles in hepatobiliary and pancreatic physiology and disease remain only partially understood [1,2,3].

These anatomical sites are frequently affected by highly aggressive malignancies [4,5]. Pancreatic ductal adenocarcinoma (PDAC) is among the most lethal cancers globally. Although the 5-year survival rate has increased from 4% to 13% over the past two decades, long-term survival remains among the lowest for all cancer types [4,5,6]. Biliary tract cancers (BTCs), including cholangiocarcinoma, although less common, are also associated with high mortality rates and a growing global burden [6,7].

In the last decade, multi-omics approaches have transformed the study of microbiota–cancer interactions by linking microbial composition, gene expression, and metabolite production to host biology. These methods have revealed how dysbiosis contributes to chronic inflammation and generates potentially carcinogenic metabolites in the biliary tract, gallbladder, and pancreas. By providing a systems-level understanding of microbial activity, multi-omics analyses have clarified mechanisms driving cancer initiation and progression and highlighted potential diagnostic biomarkers and therapeutic targets. This innovative methodology therefore offers a powerful tool for investigating and potentially modulating the role of the microbiota in oncogenesis [1,8].

Comparative analyses of bile samples using next-generation sequencing (NGS) and metagenomic approaches in benign and malignant biliary tract lesions [9,10], as well as in pancreatic diseases, have demonstrated distinct compositional patterns of the biliary microbiome, with variations in dominant bacterial phyla including Firmicutes, Bacteroidetes, Actinobacteria, and Proteobacteria. These microbial shifts may influence bile acid metabolism and the integrity of the biliary epithelial barrier, contributing to disease susceptibility and progression [10,11].

The gut–liver axis represents a major route of microbial communication, with bacteria reaching the biliary tree either by ascending from the duodenum or via the portal circulation. Disruption of this physiological balance, known as dysbiosis, is associated with several clinically significant conditions [11,12,13]. In cholelithiasis and cholecystitis, bacteria such as *Escherichia coli* and *Enterococcus* spp. produce β-glucuronidase, promoting pigmented stone formation and generating biofilms that act as scaffolds for cholesterol crystallization [1].

**Figure 1:**
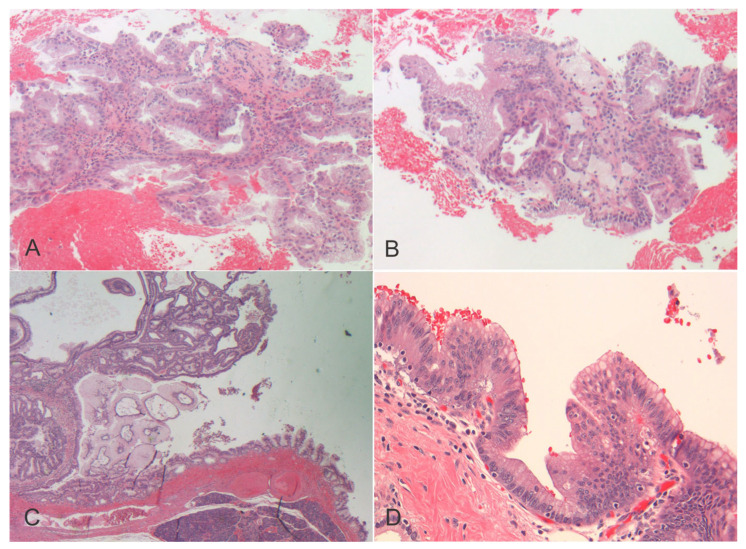
Intraductal Papillary Mucinous Neoplasm (IPMN). (**A**) IPMN, pancreatobiliary type with papillary architecture and low-grade dysplasia (magnification 50×). (**B**) IPMN, pancreatobiliary type with papillary architecture and macrophages within the fibrovascular cores (magnification 50×). (**C**) IPMN, pancreatobiliary type with complex architecture/high-grade dysplasia (magnification 25×). (**D**) IPMN, pancreatobiliary type with papillary architecture and low-grade dysplasia (magnification 200×). The microphotographs are from the personal archive of Dr. A. Thiesen.

Within this altered microenvironment, pancreatic and biliary tract precancerous lesions, identified by histopathological diagnosis—such as intraductal papillary mucinous neoplasms (IPMNs), illustrated in Figure 1, and primary sclerosing cholangitis (PSC)—are associated with alterations in the intratumoral and biliary microbiota [10,14]. Reported differences in bacterial taxa and phyla, particularly among Firmicutes and Proteobacteria, suggest a potential role of microbial communities in lesion progression and malignant transformation [2,3,10,11,12].

Collectively, these findings support the hypothesis that characterization and modulation of the biliary microbiota may have implications not only for understanding pancreaticobiliary carcinogenesis but also for prevention and therapeutic strategies in clinical practice [14,15].

Additionally, changes in the biliary, gut, and oral microbiomes contribute to infectious complications. In advanced biliary–pancreatic malignancies, such as pancreatic ductal adenocarcinoma (PDAC) and biliary tract cancer (BTC), biliary stenting is commonly used to relieve malignant biliary obstruction. However, stent placement may alter the bile microbiome, promoting colonization by antibiotic-resistant bacteria and increasing the risk of major complications including cholangitis, sepsis, and postoperative infections [3,14,15,16,17,18].

Similar microbiome–bile interactions have been reported during chemotherapy and surgical treatment for pancreatic ductal adenocarcinoma, where microbial dysbiosis and resistant bacterial strains may influence infection risk and overall clinical outcomes, including postoperative morbidity and treatment response [19,20].

Most studies examining the association between advanced pancreaticobiliary diseases, cancer, and the microbiome have been published since 2015, largely driven by the adoption of high-throughput sequencing technologies, including next-generation sequencing and metagenomic analyses. Therefore, to capture evidence generated using contemporary microbiome characterization methods, this scoping review included articles published between January 2015 and December 2025. To our knowledge, this is the first scoping review that systematically integrates biliary microbiota composition with clinical interventions (including biliary stenting and chemoprophylaxis) and emerging bioinformatics approaches in pancreaticobiliary cancers. Unlike previous reviews, which mainly focus on the gut microbiome or provide general overviews of microbiota–cancer interactions, this work specifically addresses the biliary tract and highlights clinically relevant implications, as well as current knowledge gaps and future research directions.

## 2. Materials and Methods

### 2.1. Information Sources and Search Strategy

This scoping review adhered to the PRISMA-ScR (Preferred Reporting Items for Systematic Reviews and Meta-Analyses extension for Scoping Reviews) guidelines [21], and its protocol was prospectively registered on the Open Science Framework (OSF) (DOI: 10.17605/OSF.IO/GR97B). The PRISMA-ScR checklist is provided in the Supplementary Materials (Table S1).

The search strategy was designed to identify studies investigating the role of the biliary microbiota in biliary tract and pancreatic diseases, as well as its potential implications for chemoprophylaxis and cancer management. The search was structured around three key conceptual domains: (1) microbiota and dysbiosis; (2) biliary tract and pancreatic diseases, including cancer progression; and (3) preventive or therapeutic strategies in oncologic care.

Keywords and Medical Subject Headings (MeSH) were identified through an initial screening of the literature and subsequently used to search PubMed, Scopus, and Web of Science. The search was restricted to studies published between January 2015 and December 2025. Detailed search strategies for each database are provided in Table 1.

### 2.2. Eligibility Criteria

The review process followed the PICo (Population, Interest, Context) framework, which is recommended for scoping reviews [22]. Eligible studies met the following criteria:

- Population: adult patients (≥19 years) with biliary or pancreatic tract diseases;
- Interest: biliary microbiota, carcinogenesis, and therapeutic or preventive strategies;
- Context: pathophysiology and clinical management of pancreaticobiliary oncology [21].

Only articles published in English and involving human adult populations were included. Eligible study designs comprised clinical studies, observational studies, multicenter studies, comparative studies, and phase I–IV clinical trials, including randomized controlled trials.

Exclusion criteria included in vitro experiments, animal studies, preclinical research, case reports, validation studies, study protocols, reviews, meta-analyses, guidelines, editorials, commentaries, conference abstracts, posters, books, technical reports, and preprints.

### 2.3. Research Questions

Based on emerging evidence, this scoping review addressed three main research questions:

1. How does bile microbiome dysbiosis interact with the tumor microenvironment to promote chronic inflammation, immune dysregulation, and carcinogenesis in biliary tract and pancreatic diseases?
2. What is the role of chemoprophylaxis in biliary interventions, particularly in patients undergoing biliary stenting, considering the balance between infection prevention and the induction of microbiome dysbiosis?
3. Is bile microbiota composition associated with clinical outcomes, including postoperative complications, treatment response, and cancer progression following chemotherapy and/or surgical resection?

### 2.4. Data Extraction

Two independent reviewers (PDC and NS), with expertise in virology and biostatistics, respectively, screened titles and abstracts of the retrieved records. Studies addressing the biliary tract, bile, microbiota or microbiome, pancreatic diseases, and anticancer treatments were considered potentially eligible. Full texts of relevant articles were independently assessed for inclusion. Discrepancies were resolved through discussion with a third reviewer (CMS). Data were extracted using a standardized collection form, including first author, journal, publication year, study title, database source, study aim, study design, sample size, and main findings.

## 3. Results

### 3.1. Study Selection

The database search identified a total of 242 records from Web of Science (*n* = 106), PubMed (*n* = 61), and Scopus (*n* = 75). Following the removal of 65 duplicate records, 177 studies remained for title and abstract screening. Titles and abstracts were independently screened by two reviewers (PDC and NS).

During this screening phase, 110 records were excluded because their study designs were not eligible for this scoping review, including reviews (*n* = 109) and preprints (*n* = 1). A total of 67 reports were sought for retrieval and assessed for relevance. Subsequently, 39 records were excluded because they addressed topics inconsistent with the review’s aim.

Full-text assessment was performed on 28 articles, leading to the exclusion of a further 11 studies that did not fully meet the predefined PICo criteria. Studies were excluded if they did not involve adult human populations, did not address biliary tract or pancreatic diseases, were not cancer-related, or were review articles or case reports.

**Figure 2:**
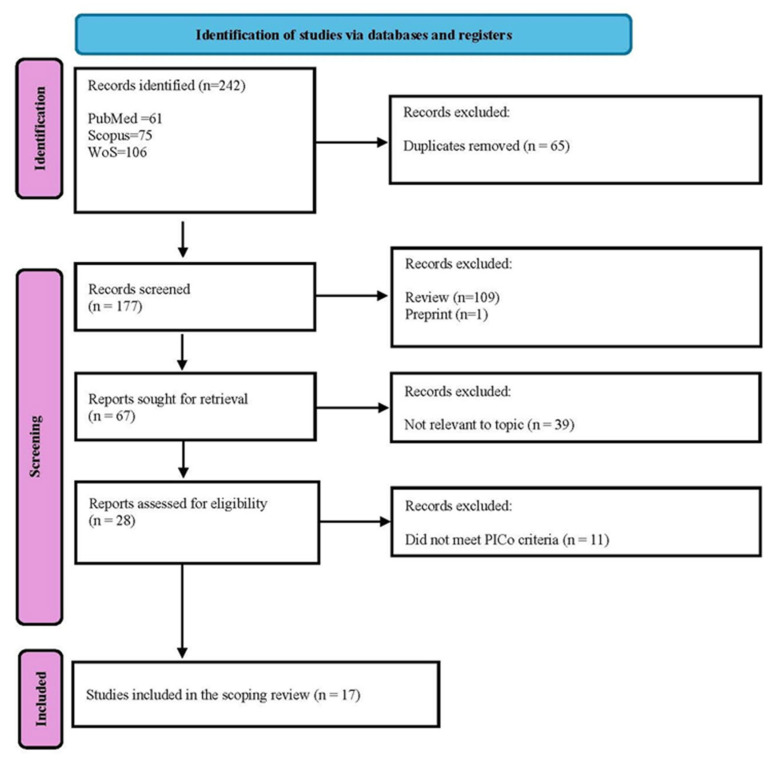
Flow diagram of the study selection process according to PRISMA 2020.

Overall, 17 studies fulfilled the eligibility criteria and were included in the scoping review (Figure 2) [18,19,23,24,25,26,27,28,29,30,31,32,33,34,35,36,37].

The characteristics of the 17 included studies are summarized in Table 2. Most studies investigated the microbiota using bile samples [19,23,24,25,26,27,28,29,30,31,32,33,34,35,37], while others analyzed fecal samples [29,30,35], tissue specimens [23,27,29], or alternative biological fluids [29].

### 3.2. Study Characteristics

#### 3.2.1. Dysbiosis Patterns and Microbial Signatures

Overall, the included studies highlighted a growing interest in the role of biliary microbiota dysbiosis in carcinogenesis, tumor progression, and treatment response in pancreaticobiliary diseases. Dysbiosis was reported in both benign conditions, such as choledocholithiasis, and malignant diseases, including distal cholangiocarcinoma (dCCA).

Avilés-Jiménez et al. [23] conducted a large multicenter case–control study comparing the microbiota of patients with extrahepatic cholangiocarcinoma (ECCA) and benign biliary diseases. The authors reported a significant enrichment of uncommon bacterial taxa in cancer patients, including Methylophilaceae and Nesterenkonia (typically associated with saline environments) and Mesorhizobium, a nitrogen-fixing bacterium usually found in plants. In addition, a greater abundance of *H. pylori* virulence genes, such as *cagA* and *vacA*, was detected in ECCA samples, suggesting a potential role of *H. pylori* in the carcinogenesis of extrahepatic cholangiocarcinoma.

Comparative studies investigating biliary microbiota in cholangiocarcinoma and choledocholithiasis have consistently identified a shared core microbial composition, predominantly comprising Proteobacteria (Pseudomonadota), Firmicutes (Bacillota), Bacteroidetes, and Actinobacteria, alongside disease-specific alterations. Chen et al. [26] reported higher microbial diversity and enrichment of rare phyla in cholangiocarcinoma, supporting malignancy-associated dysbiosis. Similarly, Park et al. [36] described a taxonomic shift in cholangiocarcinoma, characterized by increased Bacillota and a predominance of genera such as *Streptococcus* and *Veillonella*, whereas choledocholithiasis was enriched for *Escherichia*, Enterobacteriaceae, *Enterococcus*, and *Clostridium*. Consistently, Wang et al. [37] further confirmed distinct biliary microbial signatures associated with cholangiocarcinoma, highlighting alterations in both microbial composition and relative abundance, supporting the potential contribution of biliary microbiota imbalance to carcinogenesis.

Regarding pancreatic cancer and dysbiosis, Di Carlo et al. [27], using bile culture analysis, reported a high prevalence of *E. coli* and *Klebsiella* spp. in patients with pancreatic head carcinoma. These strains frequently exhibited resistance to third-generation cephalosporins, aminoglycosides, and quinolones, particularly levofloxacin. Notably, the presence of resistant strains was associated with patient survival. In a subsequent study comparing bile cultures from pancreatic and extrapancreatic tumors, the same authors observed a predominance of *E. coli*, *Klebsiella* spp., and *Pseudomonas* spp. in pancreatic cancer, together with a significantly higher prevalence of *Candida* spp. in pancreatic patients [34].

Using metagenomic analyses, Okuda et al. [29] reported the presence of bacterial genera with ≥1% relative abundance in tumor tissues and identified an increased prevalence of *Akkermansia*, a Gram-negative genus, in bile samples from pancreatic cancer patients undergoing external biliary drainage. Scheufele et al. [24] also demonstrated significant alterations in bile microbiota composition following preoperative biliary drainage in periampullary pancreatic cancer, with increased prevalence of *Enterococcus faecalis* and *Enterobacter cloacae* and a higher risk of postoperative wound infections.

Kirishima et al. [31], using metagenomic next-generation sequencing (mNGS), identified a high relative abundance of *Klebsiella*, *Veillonella*, *Acinetobacter*, *Selenomonas*, and *Paracoccus* in pancreatic adenocarcinoma; these genera were associated with a significantly poorer prognosis. Moreover, differences in the relative abundance of *Schaalia*, *Alloprevotella*, *Bilophila*, *Dialister*, *Eggerthella*, *Selenomonas*, and *Streptococcus* were observed between (IPMC) and intraductal papillary mucinous neoplasm (IPMN), suggesting microbiota-based distinctions between invasive and non-invasive lesions.

Sidiropoulos et al. [34] investigated fecal microbiota dysbiosis in patients with pancreatic adenocarcinoma and IPMN, as well as in healthy controls. At the phylum level, the microbiota was mainly composed of Bacteroidota, Firmicutes, and Proteobacteria. A comparison between healthy controls and pancreatic cancer patients revealed a modest increase in Firmicutes and Proteobacteria, while no major differences were observed between pancreatic ductal adenocarcinoma and IPMN. Additionally, Poudel et al. [32], using metagenomic analysis of bile samples, identified a predominance of genera including *Dickeya*, [*Eubacterium*] *hallii group*, *Bacteroides*, *Faecalibacterium*, *Escherichia–Shigella*, and *Ruminococcus* in pancreaticobiliary cancers compared with benign diseases, and found distinct microbiomic fingerprints that differentiate cholangiocarcinoma from pancreatic cancer.

Finally, studies by Behrens et al. [19], Goel et al. [25], and Nadeem et al. [27] evaluated the impact of neoadjuvant therapy on the microbiota of patients with pancreatic cancer. Except for Behrens et al., these studies suggested that neoadjuvant treatment may contribute to the enrichment of *Enterococcus* and *Klebsiella* and to increased antimicrobial resistance, consistent with findings reported by Di Carlo et al. in patients with pancreatic cancer [18,27,34].

#### 3.2.2. Tumor-Associated Microbial Signatures and Functional Alterations

Across the included studies, biliary dysbiosis was generally characterized by reduced microbial diversity, enrichment of potentially pro-inflammatory taxa, and altered microbial composition. Analysis of microbiota composition aimed to identify microbial biomarkers associated with pancreaticobiliary diseases [22,23,24,25,26,27,28,29,30,31,32,33,34,35,36]. Tumor-associated microbial signatures were detected at both biliary and fecal levels. Ito et al. and Sidiropoulos et al. [26,31] reported distinct microbial profiles associated with pancreaticobiliary tumors, supporting the hypothesis of microbiota–tumor interactions.

Okuda et al. [29] demonstrated that specific operational taxonomic units (OTUs) were enriched in tumor tissues and overlapped with gastric and pancreatic fluids, whereas bile samples harbored fewer OTUs, suggesting a distinct tumor-associated microbiome niche. Common limitations across studies included retrospective design, small sample sizes, heterogeneous timing of sample collection, and prior exposure to antibiotics or biliary drainage procedures.

Kirishima et al. [31] further suggested that the composition of biliary microbiota may serve as a prognostic biomarker in pancreaticobiliary tract cancers. In cholangiocarcinoma, lymph node metastasis was associated with an increased abundance of *Campylobacter, Citrobacter*, and *Leptotrichia*, whereas in pancreatic adenocarcinoma, nodal status correlated with differences in *Enterobacter*, *Hungatella*, *Mycolicibacterium*, *Phyllobacterium*, and *Sphingomonas*. Overall, pancreaticobiliary tumors exhibited dysbiotic microbial profiles compared with benign conditions, suggesting potential diagnostic and prognostic utility of biliary microbiota analysis.

Enrichment of pro-inflammatory taxa was frequently reported; however, the specific bacterial genera varied across studies and between pancreatic and extrapancreatic tumors [34]. These patterns were further modulated by clinical factors, including biliary stent placement and other interventional procedures [32].

Functional microbiome alterations were explored in a limited number of studies, primarily using predictive bioinformatics approaches. Ito et al. [30] suggested that dysbiosis may promote biliary carcinogenesis by enhancing lipopolysaccharide biosynthesis and microbial metabolic activity, contributing to inflammation, oxidative stress, and DNA damage. Similarly, Sidiropoulos et al. [35] integrated taxonomic profiling, diversity analysis, and machine learning approaches (Random Forest and LEfSe) to identify microbiota-based diagnostic signatures, although discrimination between IPMN and pancreatic ductal adenocarcinoma remained challenging.

#### 3.2.3. Impact of Clinical Interventions and Variability in Microbiota Composition

The effects of clinical interventions on biliary microbiota composition were heterogeneous. Some studies reported significant alterations in the microbiome following neoadjuvant chemotherapy [25,28], whereas others observed minimal or no effects [19]. Behrens et al. [19] found that neoadjuvant therapy did not significantly alter the biliary microbiome in patients with pancreatic cancer, suggesting that procedural factors, such as biliary drainage, exert a greater influence on microbial composition than chemotherapy itself.

Preoperative biliary drainage was consistently associated with microbial colonization and shifts toward antibiotic-resistant taxa [24]. Cohort studies have confirmed the presence of complex, heterogeneous microbial communities within the biliary–pancreatic system [27,29,34]. Seasonal variability and increasing antimicrobial resistance were reported in recent analyses, adding further complexity to microbiome interpretation [18].

#### 3.2.4. Prognostic Implications and Precancerous Lesions

Only a limited number of studies investigated the prognostic relevance of biliary microbiota composition. Kirishima et al. [31] identified associations between gallbladder microbiota profiles and prognosis in pancreaticobiliary cancers. Evidence regarding precancerous conditions was scarce; among the included studies, only Kirishima et al. examined IPMN and demonstrated differences in the relative abundance of specific genera (*Schaalia*, *Alloprevotella*, *Bilophila*, *Dialister*, *Eggerthella*, *Selenomonas*, and *Streptococcus*) between IPMN and IPMC. Representative histopathological findings of IPMN lesions are shown in Figure 1. No included studies investigated dysbiosis in other precancerous lesions of biliary tract and pancreatic cancer. Instead, most studies have focused on characterizing the microbiota in patients with choledocholithiasis, often comparing them with those in malignant conditions [23,26,33,36].

#### 3.2.5. Methodological Considerations

Culture-based approaches remain essential for studying contamination by surgical procedures and to analyze the pattern of emerging resistance and propose antibiotic prophylaxis in a particular epidemiological setting. As indicated in Table 2, most of the studies included utilized microbiome sequencing to reveal significant dysbiosis in cancer patients and to explore potential microbiota biomarkers.

Functional methodologies based on predictive bioinformatics [30], combined with machine learning approaches [35], offer additional opportunities to identify microbiota-based biomarkers by linking microbial composition to disease-related metabolic and inflammatory pathways.

## 4. Discussion

This scoping review highlights a growing body of evidence supporting a role for the microbiota in pancreatic–biliary malignancies while also revealing substantial methodological and conceptual limitations that currently hinder translation into clinical practice.

Across the included studies, dysbiosis emerged as a consistent feature of pancreatic–biliary tumours, most commonly characterized by reduced microbial diversity and a shift toward potentially pathogenic taxa. These observations align with broader oncological microbiome research, in which reduced alpha diversity has been associated with tumor-promoting microenvironments and impaired immune surveillance [38]. However, compared with the gut, the functional and mechanistic implications of biliary dysbiosis remain poorly understood [39].

A major limitation of the current evidence is the predominance of taxonomic over functional analyses. Most studies relied on 16S rRNA gene sequencing, which provides limited resolution and does not capture microbial metabolic activity or host–microbe interactions. This represents a critical gap, as microbial function—rather than mere presence—is increasingly recognized as central to oncogenic processes, including inflammation, bile acid metabolism, and immune modulation.

Considerable heterogeneity was also observed in sampling strategies, with microbiota characterized from bile, feces, and less frequently tumor tissue. These compartments are not directly comparable, and studies in other cancers have shown that tissue-resident microbiota may differ substantially from luminal communities while exerting a more direct influence on tumor biology. In this context, the study by Kirishima et al. [31] is particularly relevant, suggesting that bile microbiota composition may reflect tumor behavior and prognosis. Nevertheless, the lack of paired tissue analyses limits mechanistic interpretation.

The role of inflammation as a mediator linking microbiota and carcinogenesis in the biliary tract remains largely speculative. Although several studies have reported enrichment of pro-inflammatory taxa, direct evidence linking microbial profiles to inflammatory pathways or immune modulation remains scarce. This contrasts with gastrointestinal cancers, in which clear associations among specific microbes, chronic inflammation, and carcinogenesis have been demonstrated [40]. The absence of integrated multi-omics approaches combining microbiome, transcriptomic, and immune profiling represents an important unmet need.

The differences in microbial composition observed in the selected study potentially discriminate between cancer patients and healthy subjects but fail to distinguish precancerous lesions from cancer [31,35]. This likely reflects substantial methodological challenges, as these lesions are often microscopic, multifocal, or incidentally identified, making targeted sampling difficult. In addition, tissue-based microbiome studies in the biliary tract are particularly vulnerable to contamination due to low microbial biomass, which further limits reproducibility and confidence in the results. Consequently, the role of the microbiota in the early phases of biliary carcinogenesis remains largely unexplored. In this context, the study by Sidiropoulos et al. [35] highlights that the fecal microbiome offers the benefit of easy accessibility, as it does not require invasive procedures. However, the microbiota, as we previously reported [41], are highly influenced by multiple factors, with considerable variability among individuals, including differences between biological sexes

Clinical interventions emerged as important modifiers of the biliary microbiota. Procedures such as biliary drainage, antibiotic exposure, and systemic treatments—including neoadjuvant chemotherapy—have been shown to alter microbial composition and are major confounding factors [20,25,28]. Conflicting findings on the impact of chemotherapy underscore the complexity of disentangling disease-related changes from treatment-induced effects. Similar challenges have been reported in other cancers, where therapy-related microbiome shifts can both influence and obscure associations with clinical outcomes [42].

Another underexplored aspect is the burden of antibiotic-resistant bacteria within the biliary microbiota. Emerging evidence highlights the clinical relevance of resistance patterns, particularly in perioperative management and infectious complications. The biliary microbiome itself may serve as a reservoir of resistance genes, potentially influencing therapeutic outcomes. Advanced non-invasive approaches, such as metabolomic profiling of fecal samples, are essential for comprehensively characterizing these features [43].

The prognostic significance of biliary microbiota composition remains under investigation. Preliminary data suggest associations with survival and disease progression, but available studies are limited by small sample sizes and retrospective designs, underscoring the need for validation in larger, prospective cohorts.

Overall, the findings of this scoping review suggests a potential role of the biliary microbiota in pancreatic–biliary tumor biology, although the available evidence is predominantly descriptive and exploratory.

Future research should prioritize (i) standardization of sampling and analytical methodologies; (ii) integration of multi-omics approaches to elucidate mechanisms; (iii) longitudinal study designs to capture temporal dynamics; (iv) evaluation of microbiota as a biomarker of treatment response; and (v) exploration of microbiota-targeted strategies, including antibiotic stewardship and microbiome modulation.

## 5. Conclusions

Specific microbial signatures influence patient responses to cancer immunotherapy. Greater microbial diversity has been associated with improved clinical outcomes, whereas antibiotic exposure may compromise treatment efficacy by disrupting microbial homeostasis. Moreover, specific microbial signatures appear to modulate immunotherapeutic responses, supporting a potential causal role of the microbiota and opening new avenues for personalized therapeutic strategies [44].

In parallel, biliary tract and pancreatic cancers have been associated with microbial dysbiosis, which may contribute to tumour development, progression, and treatment response. However, current evidence remains largely descriptive, providing limited mechanistic insight into the functional role of microbiota and its causal relationship with disease outcomes. A deeper understanding of host–microbiome interactions may open new avenues for diagnostic, prognostic, and therapeutic applications, including microbiome-based biomarkers and targeted modulation strategies aimed at improving patient outcomes. Future studies integrating standardized methodologies and multi-omics approaches will be essential to effectively translate these findings into clinical practice. Advancing this field will require more robust, standardized, and mechanistically oriented studies, supported by close multidisciplinary collaboration among oncologists, gastroenterologists, surgeons, microbiologists, pathologists, bioinformaticians, and translational researchers to better elucidate microbiota–host interactions and their clinical implications.

## Figures and Tables

**Table 1:** Strings used in the search for each database.

Database	Search Terms	Records Retrieved
PubMed	((biliary microbiome [Title/Abstract] OR bile microbiota [Title/Abstract] OR dysbiosis [Title/Abstract]) AND (biliary tract disease [Title/Abstract] OR biliary tract cancer [Title/Abstract] OR cholangiocarcinoma [Title/Abstract] OR pancreatic cancer [Title/Abstract] OR precancerous lesions [Title/Abstract]) AND (chemoprophylaxis [Title/Abstract] OR treatment [Title/Abstract])) AND ((“2015/01/01”[Date—Publication]: “2025/12/31”[Date—Publication]))	60
Scopus	TITLE-ABS-KEY (bile microbiota* Biliary tract* cancer* OR therapy OR chemoprophylaxis) AND PUBYEAR > 2014 AND PUBYEAR < 2026	75
WoS	(“biliary microbiome” OR “bile microbiota” OR dysbiosis) AND (“biliary tract disease” OR “biliary tract cancer” OR cholangiocarcinoma OR “pancreatic cancer” OR “precancerous lesions”) AND (chemoprophylaxis OR treatment) AND (2015–2025)	106

**Table 2:** Details of the 17 Studies selected for this Scoping Review (mNGS: metagenomic next-generation sequencing; CCA: cholangiocarcinoma; PDA: pancreatic ductal adenocarcinoma; BTPC: Biliary Tract and Pancreatic Cancer; IPMN: intraductal papillary mucinous neoplasms; MDR: multidrug-resistant; OTUs: operational taxonomic units.

First Author/Year	Lesion Type/Sample	Study Design/n°	Sample/Microbiological Methodology	Key Findings
Avilés-Jiménez, F., 2016 [23]	Extrahepatic CCA vs. benign biliary pathology (BBP)	multicentre case–control/200	tissue and bile/mNGS	Distinct microbiotas are present in ECCA and BBP. *H. pylori* virulence genes in ECCA.
Scheufele, F., 2017 [24]	Preoperative biliary drainage (PBD) in periampullary tumors	case–control/290	Bile/culture	PBD favors biliary microbiome contamination; PBD shifts microbiome toward more resistant bacteria; PBD increases wound infections.
Goel, N., 2019 [25]	Neoadjuvant therapy (NT) in PDA	case–control/172	Bile/culture	NT alters bile microbiome, favour resistance to cephalosporins, NT pts had more *Enterococcus*/*Klebsiella.*
Chen, B., 2019 [26]	Distal CCA vs. gallstones	cross-sectional/68	Bile/mNGS	CCA had less diversity. Proteobacteria/Firmicutes dominate in CCA.
Di Carlo, P., 2019 [27]	BTPC vs. benign diseases	cohort/152	Bile/culture	*E. coli* and *K. pneumoniae* were more linked with pancreatic cancer and showed resistance. *E. coli* presence linked to lower survival.
Nadeem, S.O., 2021 [28]	NT of PDAC and biliary microbiome	cohort/168	Bile/culture	NT exposure changes resistance profiles. NT favors resistant bacteria.
Okuda, S., 2022 [29]	BTPC and external biliary drainage (EBD)	cross-sectional/15	tissues and fluids/mNGS	*Akkermansia* bacteria were more abundant in the bile of patients with EBD. At the genus level bacteria showed 1% or more relative abundance in tumour tissue.
Ito, Z., 2022 [30]	Biliary Tract Cancer (BTC)	case–control/51	bile and fecal/culture and mNGS	Higher Enterobacteriaceae abundance and a lower Clostridia in BTC. Bile OTUs matched with faecal OTUs in BTC.
Kirishima, M., 2022 [31]	BTPC and Pancreatic Cystic Lesion (IPMN)	case–control/244	Bile/mNGS	High relative abundance of *Enterococcus*, *Eggerthella*, *Klebsiella*, *Corynebacterium*, *Moraxella*, *Hungatella*, *Paracoccus*, *Dermacoccus*, *Citrobacter*, *Lawsonella* and *Pseudoxanthomonas* in cancer pts showed a significantly poor prognosis.
Behrens, S., 2023 [19]	PDAC and NT	retrospective/346	Bile/culture	NT does not alter bile microbiome.
Poudel, S.K., 2023 [32]	BTPC vs. benign disease	case–control/46	Bile/mNGS	*Enterococcus*, *Streptococcus*, and *Bacteroides* higher in cancer.
Azimirad, M., 2023 [33]	Gallstones and hepatobiliary disease	cross-sectional/15	Bile/culture and mNGS	Higher diversity in biliary bacterial population of pts with gallstones. *Clostridium* spp. and *Peptoclostridium* spp. mainly in bile of symptomatic patients.
Di Carlo, P., 2024 [34]	Pancreatic vs. extra-pancreatic tumors	case–control/145	Bile/culture	Distinct bile microbiota; *Enterococcus* spp. common; *Candida* spp. more in PC; Alcaligenes faecalis potential marker for extra-pancreatic tumors.
Sidiropoulos, T., 2024 [35]	PDAC, IPMN and health control	case–control/33	Fecal/mNGS	Fecal dysbiosis; *Fusobacterium* and *Escherichia-Shigella* increased. Fecal microbiome as a potential non-invasive biomarker.
Park, W., 2025 [36]	CCA vs. benign biliary disease	case–control/25	Bile/mNGS	Dysbiosis in CCA; Bacillota and *Streptococcus* spp. Predominance.
Wang, J., 2025 [37]	Obstructive jaundice in benign and malignancy biliary disease	cross-sectional/42	Bile/mNGS	Microbiota and biochemical analytes vary by obstruction cause. These microbial patterns may offer new diagnostic markers and insight into biliary disease mechanisms.
Di Carlo, P., 2025 [18]	BTPC, seasonal variation	retrospective/149	Bile/culture	Isolates showed seasonal variation; rising MDR bacteria in warmer months.

## Data Availability

No new data were generated for this study. All analyzed data are included in the article.

## Supplementary Materials

The following supporting information can be downloaded at: https://www.mdpi.com/article/10.3390/cancers18121875/s1, Table S1: Preferred Reporting Items for Systematic reviews and Meta-Analyses extension for Scoping Reviews (PRISMA-ScR) Checklist.

## Abbreviations

The following abbreviations are used in this manuscript:

BTPD	Biliary Tract and Pancreatic Cancer
PICo	Patient, Intervention, Comparison, outcome
CCA	Cholangiocarcinoma
PDAC	Pancreatic ductal adenocarcinoma
IPMN	Intraductal Papillary Mucinous Neoplasm
IPMC	Intraductal Papillary Mucinous Carcinoma
mNGS	metagenomic next-generation sequencing
NT	Neoadjuvant therapy
OTUs	operational taxonomic units
