# Supplementary material for: Biliary Tract and Pancreatic Cancer (BTPC) in Adult Patients: The Role of the Biliary Microbiota in Cancer and Therapeutic Strategies—A Scoping Review
Source: Cancers (Basel). 2026 Jun 8;18(12):1875. doi: 10.3390/cancers18121875 (PMC13297221; doi:10.3390/cancers18121875)
Supplement: Supplementary file 1 [file cancers-18-01875-s001.zip › cancers-4324017-supplementary.pdf]

Preferred Reporting Items for Systematic reviews and Meta-Analyses extension for Scoping Reviews (PRISMA-ScR) Checklist

SECTION	ITEM	PRISMA-ScR CHECKLIST ITEM	REPORTED ON PAGE #
**TITLE**			
Title	1	Identify the report as a scoping review.	The type of review is explicitly identified in the title as a scoping review. (page 1)
**ABSTRACT**			
Structured summary	2	Provide a structured summary that includes (as applicable): background, objectives, eligibility criteria, sources of evidence, charting methods, results, and conclusions that relate to the review questions and objectives.	The abstract provides a structured summary including background, objectives, methods, sources of evidence, results, and conclusions related to the scope and aims of the review. (pages 1-2)
**INTRODUCTION**			
Rationale	3	Describe the rationale for the review in the context of what is already known. Explain why the review questions/objectives lend themselves to a scoping review approach.	The introduction describes the current knowledge on biliary and pancreatic tract microbiota and explains the rationale for conducting a scoping review to map available evidence and identify knowledge gaps. (pages 3-4)
Objectives	4	Provide an explicit statement of the questions and objectives being addressed with reference to their key elements (e.g., population or participants, concepts, and context) or other relevant key elements used to conceptualize the review questions and/or objectives.	The objectives of the review are explicitly stated, focusing on the role of biliary microbiota in pancreaticobiliary carcinogenesis, diagnosis, prognosis, and therapeutic strategies, framed according to the PICo approach. (page 2)
**METHODS**			
Protocol and registration	5	Indicate whether a review protocol exists; state if and where it can be accessed (e.g., a Web address); and if available, provide registration information, including the registration number.	The review protocol was prospectively registered on the Open Science Framework (OSF), and registration details including the DOI are reported. (page 4)
Eligibility criteria	6	Specify characteristics of the sources of evidence used as eligibility criteria (e.g., years considered, language, and publication status), and provide a rationale.	Eligibility criteria are defined according to population, interest, and context (PICo), including years of publication, language restrictions, study

St. Michael's
Inspired Care.
Inspiring Science.

SECTION	ITEM	PRISMA-ScR CHECKLIST ITEM	REPORTED ON PAGE #
			designs, and explicit inclusion and exclusion criteria. (pages 5)
Information sources*	7	Describe all information sources in the search (e.g., databases with dates of coverage and contact with authors to identify additional sources), as well as the date the most recent search was executed.	The information sources used for the search are described, including PubMed, Scopus, and Web of Science, with specification of the temporal coverage and the date range of eligible publications. (pages 4)
Search	8	Present the full electronic search strategy for at least 1 database, including any limits used, such that it could be repeated.	The full electronic search strategy is reported, including keywords, Boolean operators, and limits, and is presented in detail for each database. (Page 4-5)
Selection of sources of evidence†	9	State the process for selecting sources of evidence (i.e., screening and eligibility) included in the scoping review.	The process for selecting sources of evidence is described, including independent screening of titles, abstracts, and full texts by two reviewers, with resolution of disagreements through discussion with a third reviewer. (pages 5-6)
Data charting process‡	10	Describe the methods of charting data from the included sources of evidence (e.g., calibrated forms or forms that have been tested by the team before their use, and whether data charting was done independently or in duplicate) and any processes for obtaining and confirming data from investigators.	Data charting was conducted using a standardized extraction form, with data independently extracted by two reviewers and discrepancies resolved by consensus. (page 5)
Data items	11	List and define all variables for which data were sought and any assumptions and simplifications made.	The data items extracted from each study are listed and include publication details, study design, sample characteristics, microbiological methods, and main findings relevant to the review questions. (page 5)
Critical appraisal of individual sources of evidence§	12	If done, provide a rationale for conducting a critical appraisal of included sources of evidence; describe the methods used and how this information was used in any data synthesis (if appropriate).	Critical appraisal of individual sources of evidence was not performed, as it is not mandatory for scoping reviews and was not

SECTION	ITEM	PRISMA-ScR CHECKLIST ITEM	REPORTED ON PAGE #
			consistent with the exploratory aims of the study. (Not performed)
Synthesis of results	13	Describe the methods of handling and summarizing the data that were charted.	The methods used to synthesize the charted data are described, consisting of a descriptive and narrative synthesis organized by thematic areas. (page 9-11)
**RESULTS**			
Selection of sources of evidence	14	Give numbers of sources of evidence screened, assessed for eligibility, and included in the review, with reasons for exclusions at each stage, ideally using a flow diagram.	The number of records identified, screened, assessed for eligibility, and included in the review, along with reasons for exclusion, are reported and summarized in a PRISMA 2020 flow diagram. (pages 5-6)
Characteristics of sources of evidence	15	For each source of evidence, present characteristics for which data were charted and provide the citations.	The main characteristics of the included studies, such as study design, sample type, microbiological methodology, and key findings, are presented in a summary table. (pages 6-8)
Critical appraisal within sources of evidence	16	If done, present data on critical appraisal of included sources of evidence (see item 12).	No critical appraisal of included sources of evidence was performed. (Not performed)
Results of individual sources of evidence	17	For each included source of evidence, present the relevant data that were charted that relate to the review questions and objectives.	The results of individual studies are described in detail, organized by disease type, sampling strategy, microbiological findings, and clinical implications. (pages 7-11)
Synthesis of results	18	Summarize and/or present the charting results as they relate to the review questions and objectives.	The results are synthesized narratively and thematically, highlighting patterns of dysbiosis, microbial signatures, and associations with clinical outcomes. (pages 9-11)
**DISCUSSION**			
Summary of evidence	19	Summarize the main results (including an overview of concepts, themes, and types of evidence	The discussion summarizes the main findings of the review, linking available

SECTION	ITEM	PRISMA-ScR CHECKLIST ITEM	REPORTED ON PAGE #
		available), link to the review questions and objectives, and consider the relevance to key groups.	evidence to the review objectives and contextualizing results within current knowledge of pancreaticobiliary microbiota and cancer. (pages 11-13)
Limitations	20	Discuss the limitations of the scoping review process.	The limitations of the scoping review are discussed, including heterogeneity of study designs, variability in sampling and methodologies, and the predominantly descriptive nature of the available evidence. (pages 12-13)
Conclusions	21	Provide a general interpretation of the results with respect to the review questions and objectives, as well as potential implications and/or next steps.	The conclusions provide an overall interpretation of the findings, their implications for research and clinical practice, and directions for future studies. (pages 13)
**FUNDING**			
Funding	22	Describe sources of funding for the included sources of evidence, as well as sources of funding for the scoping review. Describe the role of the funders of the scoping review.	The sources of funding for the scoping review are reported, and it is stated that the study received no external funding. (page 13)

JBI = Joanna Briggs Institute; PRISMA-ScR = Preferred Reporting Items for Systematic reviews and Meta-Analyses extension for Scoping Reviews.
* Where *sources of evidence* (see second footnote) are compiled from, such as bibliographic databases, social media platforms, and Web sites.
† A more inclusive/heterogeneous term used to account for the different types of evidence or data sources (e.g., quantitative and/or qualitative research, expert opinion, and policy documents) that may be eligible in a scoping review as opposed to only studies. This is not to be confused with *information sources* (see first footnote).
‡ The frameworks by Arksey and O'Malley (6) and Levac and colleagues (7) and the JBI guidance (4, 5) refer to the process of data extraction in a scoping review as data charting.
§ The process of systematically examining research evidence to assess its validity, results, and relevance before using it to inform a decision. This term is used for items 12 and 19 instead of "risk of bias" (which is more applicable to systematic reviews of interventions) to include and acknowledge the various sources of evidence that may be used in a scoping review (e.g., quantitative and/or qualitative research, expert opinion, and policy document).

From: Tricco AC, Lillie E, Zarin W, O'Brien KK, Colquhoun H, Levac D, et al. PRISMA Extension for Scoping Reviews (PRISMAScR): Checklist and Explanation. Ann Intern Med. 2018;169:467–473. doi: 10.7326/M18-0850.
